# Photosynthesis in rice is increased by CRISPR/Cas9-mediated transformation of two truncated light-harvesting antenna

**DOI:** 10.3389/fpls.2023.1050483

**Published:** 2023-01-19

**Authors:** Daniel Caddell, Noah J. Langenfeld, Madigan JH. Eckels, Shuyang Zhen, Rachel Klaras, Laxmi Mishra, Bruce Bugbee, Devin Coleman-Derr

**Affiliations:** ^1^ Plant Gene Expression Center, United States Department of Agriculture - Agricultural Research Service (USDA ARS), Albany, CA, United States; ^2^ Plant and Microbial Biology Department, University of California at Berkeley, Berkeley, CA, United States; ^3^ Department of Plants, Soils, and Climate, Utah State University, Logan, UT, United States; ^4^ Department of Horticultural Sciences, Texas A&M University, College Station, TX, United States

**Keywords:** truncated light antenna, photosynthesis, chlorophyll, CRISPR/Cas9, rice

## Abstract

Plants compete for light partly by over-producing chlorophyll in leaves. The resulting high light absorption is an effective strategy for out competing neighbors in mixed communities, but it prevents light transmission to lower leaves and limits photosynthesis in dense agricultural canopies. We used a CRISPR/Cas9-mediated approach to engineer rice plants with truncated light-harvesting antenna (TLA) via knockout mutations to individual antenna assembly component genes CpSRP43, CpSRP54a, and its paralog, CpSRP54b. We compared the photosynthetic contributions of these components in rice by studying the growth rates of whole plants, quantum yield of photosynthesis, chlorophyll density and distribution, and phenotypic abnormalities. Additionally, we investigated a Poales-specific duplication of CpSRP54. The Poales are an important family that includes staple crops such as rice, wheat, corn, millet, and sorghum. Mutations in any of these three genes involved in antenna assembly decreased chlorophyll content and light absorption and increased photosynthesis per photon absorbed (quantum yield). These results have significant implications for the improvement of high leaf-area-index crop monocultures.

## 1 Introduction

In plants, pigments such as chlorophyll and carotenoids harvest light and transfer the energy toward reaction centers, where it is converted to chemical energy via photochemical charge separation, initiating photosynthesis. In full sunlight, plants absorb more light than can be used for photosynthesis. This overaccumulation enables a selective advantage in natural communities, whereby plants with large light-harvesting antenna complexes can outcompete neighboring plants for sunlight, which is a major growth-limiting factor in most environments. Prevention of weed establishment in agricultural settings is often accomplished through herbicidal or management practices, such as planting in dense monocultures. The absorption of excess photons by upper leaves reduces the amount of light within the canopy that is used for photosynthesis, and can negatively impact plant yields (Bugbee, 1992). Recent studies have suggested that mutations that reduce the size of light-harvesting antenna complexes, producing mutants commonly referred to as truncated light antenna (TLA) mutants, may improve quantum yield of PSII by increasing light penetration deeper into the canopy, particularly under high density, high light conditions (Gu et al., 2017a; Gu et al., 2017b; Kirst et al., 2017; Kirst et al., 2018).

Chloroplast light-harvesting antenna assembly relies on cytosolic synthesis of nuclear-encoded light-harvesting chlorophyll a/b binding proteins (LHCPs). To function, LHCPs must be imported into the chloroplast, transported, and ultimately assembled into the thylakoid membrane. This process is mediated by the chloroplast signal recognition particle (CpSRP) pathway (Ziehe et al., 2017). In the stroma, CpSRP43 and CpSRP54 form a heterodimer that binds LHCPs, forming a transit complex (DeLille et al., 2000; Tu et al., 2000; Jonas-Straube et al., 2001; Hermkes et al., 2006). This complex is important for preventing aggregation of hydrophobic LHCPs prior to insertion into the thylakoid membrane (Payan and Cline, 1991; Li et al., 1995; Schuenemann et al., 1998; Klimyuk et al., 1999; Tu et al., 1999). At the thylakoid membrane, the CpSRP receptor, CpFTSY, binds the transit complex (Kogata et al., 1999; Tu et al., 1999; Stengel et al., 2007; Chandrasekar et al., 2008) and positions it such that LHCPs can be integrated into the thylakoid membrane by the insertase ALBINO3 (ALB3) (Moore et al., 2000; Yuan et al., 2002; Moore et al., 2003).

Reducing light harvesting pigment concentration has been shown to improve photosynthesis in microalgae (Nakajima and Ueda, 1997; Nakajima et al., 2001) and cyanobacteria (Kirst et al., 2014). In plants, mutants of the CpSRP pathway (TLA mutants) have been identified in genetic screens by pale green leaf phenotypes that correspond to higher chlorophyll a/b ratios (Liu et al., 2016; Kirst et al., 2017; Kirst et al., 2018). Multiple studies have demonstrated trait alterations in TLA mutant plants with the potential for agronomic benefit, with particular focus on plants with mutations in CpSRP43 (TLA3 mutants) and CpSRP54 (TLA4 mutants). For example, the Arabidopsis TLA3 mutant *chaos* showed significantly higher tolerance to photooxidative stress in lab and field conditions (Klenell et al., 2005), and CpSRP43 knockdown in *Nicotiana tobacum* was recently demonstrated to enhance leaf-to-stem ratio and plant biomass (Kirst et al., 2018). Similarly, a TLA3 mutation in rice reduced plant height and tillering with no loss of grain mass (Lv et al., 2015), and three distinct TLA4 mutant alleles have been identified in rice that cause reduced tillering with no loss of grain weight (Zhang et al., 2013; Liu et al., 2016). In the Poales family, mutations to paralogous proteins CpSRP54a and CpSRP54b (creating TLA4 and TLA4L mutants, respectively) also caused plants to display a pale-green phenotype, although TLA4L mutants died before reaching maturity (Shi et al., 2020). In contrast to potential benefits associated with TLA3 and TLA4 mutants, plants lacking CpFTSY (TLA2 mutants) or ALB3 have been shown to suffer significant pleiotropic effects on growth and development. For example, Arabidopsis *cpftsY* and *alb3* mutants (Durrett et al., 2006; Asakura et al., 2008), and a maize *cpftsY* null-mutant (Asakura et al., 2004) had pleiotropic defects associated with broad roles in thylakoid biogenesis in addition to a substantial reduction in chlorophyll.

Previous research has indirectly compared various molecular characteristics of TLA mutants in Arabidopsis (Amin et al., 1999; Asakura et al., 2008; Walter et al., 2015). However, direct comparisons of potential agronomic benefits between TLA mutants in crop plants have not been performed, in part because they have been independently identified and characterized in different genetic backgrounds, which can prevent the ability to distinguish phenotypic differences due to broad genotypic contributions from specific TLA mutations. Gene engineering mediated by CRISPR/Cas9 now provides the ability to compare gene function in near-isogenic backgrounds, allowing for a direct dissection of the contribution of each TLA mutation to photosynthesis and fitness phenotypes. In this study, we used CRISPR/Cas9 to create three independent mutant lines, each with a single null mutation in predicted components of the rice CpSRP pathway; *CpSRP43* (TLA3), *CpSRP54a* (TLA4), and *CpSRP54b* (TLA4L), and assessed their potential agronomic benefits.

## 2 Materials and methods

### 2.1 Plasmid construction

Assembly of CRISPR/Cas9 T-DNA vectors was performed as described previously (Lowder et al., 2015). In short, sgRNA oligo pairs targeting *CpSRP43* (LOC_Os03g03990), *CpSRP54a* (LOC_Os11g05552), and *CpSRP54b* (LOC_Os11g05556) were designed and annealed (**Table 1**). Annealed oligos with a single target near the N-terminal of CpSRP54a or CpSRP54b were cloned into the gateway entry vector pYPQ141C (Lowder et al., 2015) to create 1-TLA4 and 1-TLA4L. Annealed oligos for golden gate assembly were cloned into the golden gate recipient vectors: pYPQ131C, pYPQ132C, or pYPQ133C (Lowder et al., 2015). Proper ligation of oligos was confirmed at all stages by Sanger sequencing with either primer M13-F (for pYPQ141C) or pTC14-F2 (for pYPQ131C, pYPQ132C, and pYPQ133C). See **Table 2** for the sequences of primers used in this study. The following golden gate assemblies were also performed: assembly of two sgRNAs targeting the N- and C-terminal regions of CpSRP43, CpSRP54a, or CpSRP54b into pYPQ142 (Lowder et al., 2015) to create 2-TLA3, 2-TLA4, and 2-TLA4L, respectively, and assembly of three sgRNAs targeting near the N- and C-terminal of CpSRP54a and the C-terminal of CpSRP54b into pYPQ143 (Lowder et al., 2015) to create TLA4/TLA4L. Gateway assembly of the sgRNA entry vectors, the Cas9 entry vector pYPQ167 (Lowder et al., 2015), and the gateway-compatible destination vector Ubi-CAMBIA-1300 (Chern et al., 2001) were performed using LR clonase II enzyme (Thermo Fisher Scientific, Waltham, MA), to generate the final CRISPR/Cas9 T-DNA vectors Ubi-2-TLA3, Ubi-1-TLA4, Ubi-2-TLA4, Ubi-1-TLA4L, Ubi-2-TLA4L, and Ubi-TLA4/TLA4L. Proper assembly was verified by restriction digestion and sequencing with Ubi-pro-F and Cas9-seq-R primers (**Table 2**).

**Table 1:** sgRNA oligo pairs designed to produce CRISPR/Cas9-based knockouts of *CpSRP43*, *CpSRP54a*, and *CpSRP54b*.

Construct name	sgRNA Oligo Pair Sequence 5’ – 3’	Target
1-TLA3	gtgtgCGAGCCTTCGTGGATCCCGG	*CpSRP43* N-terminal
	aaacCCGGGATCCACGAAGGCTCGc	
1-TLA4	gtgtgTCTTATAAGAGGAGTCCGAC	*CpSRP54a* N-terminal
	aaacGTCGGACTCCTCTTATAAGAc	
1-TLA4L	gtgtgGTAGGCACTGATGTGATTCG	*CpSRP54b* N-terminal
	aaacCGAATCACATCAGTGCCTACc	
2-TLA3	gtgtgCCACTCGACGAGGTACTCCG	*CpSRP43* N-terminal
	aaacCGGAGTACCTCGTCGAGTGGc	
	gtgtgTTCGGCGTCGACGTTCTCCG	*CpSRP43* C-terminal
	aaacCGGAGAACGTCGACGCCGAAc	
2-TLA4	gtgtgGCCACGCAGCTTGTTCCAGG	*CpSRP54a* N-terminal
	aaacCCTGGAACAAGCTGCGTGGCc	
	gtgtgGCATTTGTAGATATGATGGT	*CpSRP54a* C-terminal
	aaacACCATCATATCTACAAATGCc	
2-TLA4L	gtgtgTCCCCGCAGCTTGTTCCACG	*CpSRP54b* N-terminal
	aaacCGTGGAACAAGCTGCGGGGAc	
	gtgtgCCGCTGGTACTGGAAAGCGA	*CpSRP54b* C-terminal
	aaacTCGCTTTCCAGTACCAGCGGc	
TLA4/TLA4L	gtgtgTCTTATAAGAGGAGTCCGAC	*CpSRP54a* N-terminal
	aaacGTCGGACTCCTCTTATAAGAc	
	gtgtgGCTCTTCTGATATCCCGCAT	*CpSRP54a* C-terminal
	aaacATGCGGGATATCAGAAGAGCc	
	gtgtgCCGCTGGTACTGGAAAGCGA	*CpSRP54b* N-terminal
	aaacTCGCTTTCCAGTACCAGCGGc	

Systems designed to target one, two, or three loci can be differentiated by the number of sgRNA sequences listed under the construct name.

**Table 2:** Primers used in this study. Both sequencing and RT-PCR primers are listed with corresponding purposes.

Primer Name	Sequence 5’ – 3’	Purpose
M13 F	TGTAAAACGACGGCCAGT	Sequencing of pYPQ141C vectors
pTC14-F2	CAAGCCTGATTGGGAGAAAA	Sequencing of pYPQ131C, pYPQ132C, and pYPQ133C vectors
Ubi-pro-F	TTGTCGATGCTCACCCTGTTGTTT	Forward sequencing of Ubi-CAMBIA-1300 vectors
Cas9-seq-R	ACCGTCAATGTAACCGGCGTAG	Reverse sequencing of Ubi-CAMBIA-1300 vectors
TLA4-F	AGAGGAGTCCGACCGGAACAGC	RT-PCR of *CpSRP54a*
TLA4-R	TTCCCAACACCTTGCAGGCCTG	
TLA4L-F	AGCCAATTGGTTGCGCAGCTCT	RT-PCR of *CpSRP54b*
TLA4L-R	CTTTCCAGTACCAGCGGCAGCC	
Actin-F	GTCCTCTTCCAGCCTTCCTT	RT-PCR of Actin-1 gene as internal control
Actin-R	GCGACCACCTTGATCTTCAT	

### 2.2 Rice transformation

Transformation of *Agrobacterium tumefaciens* strain EHA105 with plasmids Ubi-2-TLA3, Ubi-1-TLA4, Ubi-2-TLA4, Ubi-1-TLA4L, Ubi-2-TLA4L, or Ubi-TLA4/TLA4L was performed by electroporation using the Gene Pulser Xcell (Bio-Rad), per the manufacturer’s instructions. Rice transformation was performed at the University of California, Davis Rice Transformation Facility using *A. tumefaciens* strain EHA105 to infect rice calli (*Oryza sativa* ssp. japonica L., cv. Kitaake). Selection of transformants carrying the transgenes was performed using hygromycin as a selectable marker and later confirmed by PCR with transgene specific primers Ubi-pro-F and Cas9-seq-R (**Table 2**). Identification of successful CRISPR/Cas9-mediated indels was determined by PCR using primers flanking the target cut site. Indels were further validated by Sanger sequencing. All plants used in this study were homozygous TLA mutants unless otherwise specified as null mutants (n).

### 2.3 Plant growing conditions and phenotyping

Rice plants were grown in the greenhouse with 16-h day and 8-h night cycle, 28°C day and 26°C night temperature, and 50-60% relative humidity. Plants were grown in a peat-based soilless media and watered daily with a complete nutrient solution (Zhen and Bugbee, 2020). The solution was supplemented with 20 ppm ethylenediamine-N,N′-bis(2-hydroxyphenylacetic acid) (EDDHA) chelated iron to minimize any possible iron chlorosis. Photosynthetic rate was measured using a portable photosynthesis system (model LI-6800, LICOR Biosciences, Lincoln, NE) with a fluorometer head attached (model LI-6800-01A, LICOR Biosciences). The chamber settings held constant across measurements were: 25°C air temperature, 150 µmol s^-1^ flow rate, 5000 rpm fan speed, 50% relative humidity, 90% red photon fraction, and 10% blue photon fraction. Light response curves (LRCs) were generated by measuring assimilation rates stepwise at 400, 100, 0, 50, 200, 400, 800, 1400, and 2000 µmol photons m^-2^ s^-1^ using the built-in light response curve program. A minimum chamber stabilization time of 10 minutes was set, and carbon dioxide and water infrared gas analyzers were matched following the logging of each measurement. LRCs were run first at 400 ppm CO_2_ and then at 1200 ppm CO_2_ without removing the leaf from the chamber. All photosynthetic rate measurements were collected on rice plants ranging from 33 to 35 d old.

Photon absorbance was calculated by subtraction from measurements of transmission and calculated reflectance. Photon transmission was measured after placing the leaf over quantum sensor (model LI-190R, LICOR Biosciences). The transmitted fraction of photons was multiplied by 2 to account for an equal number of reflected and transmitted photons (Jacquemoud and Ustin, 2019). Photosynthetic rates were then divided by the absorbed photon flux to calculate photosynthetic rate per photon absorbed. Chlorophyll content was measured using an optical chlorophyll meter (Model MC-100, Apogee Instruments, Logan, UT) using generic coefficients (Parry and Bugbee, 2017). Laboratory measurements were made on nine leaves and averaged. To confirm optical chlorophyll measurements, 0.05 g of tissue was cut from the newest fully expanded leaf of mature rice plants and ground to a fine powder in liquid N_2_. Photosynthetic pigment was extracted using 80% acetone and incubated in the dark at room temperature for 24-h. Absorbance measurements at 663.2 nm, 646.8 nm, and 470 nm were recorded using a Shimadzu UV-1280 spectrophotometer. Estimates of chlorophyll and carotenoid content were calculated with the equations previously described by Wellburn (1994).

### 2.4 CpSRP54 alignments and phylogenetic tree

Protein blast was performed using Arabidopsis CpSRP54 (At5g03940) as an input in phytozome v12.1 against other genomes, retrieving 188 hits. Because plants have both an SRP54 and CpSRP54 system, we used ChloroP v1.1 (Emanuelsson et al., 1999) to predict which proteins were chloroplast localized, reducing our protein lists to 60 hits. Of those 60, 32 genes that were predicted to encode the SRP receptor (CpFTSY) were removed from the list, leaving 28 genes predicted to encode CpSRP54 across 21 species. CpSRP54 proteins were aligned using the MUSCLE alignment algorithm, with 100 iterations. The phylogenetic tree was built using the Jukes-cantor neighbor joining method, with 1,000 bootstraps. The CpSRP pathway functional gene network predictions were made using RiceNet v2 (Lee et al., 2015) with default parameters.

### 2.5 TLA gene expression

Leaf tissue from the youngest fully expanded leaves of three independent TLA4 mutant lines and Kitaake WT was collected from four-week-old plants (five plants per line) and RNA was extracted using a Qiagen Plant RNA extraction kit. RNA was converted to cDNA using a cDNA synthesis kit, and quantitative RT-PCR was performed using primers specific to *CpSRP54a* (TLA4-F/R) or *CpSRP54b* (TLA4L-F/R) (**Table 2**). Actin-1 (LOC_Os03g50885) gene expression was amplified as a control using the primers Actin-F/R (**Table 2**).

### 2.6 Statistical analyses

Statistical tests were performed using a one-way analysis of variance (ANOVA) test. Statistically significant results were followed up with a Tukey’s honest significance *post-hoc* test using R (Version 3.6.1, R Core Team). Significance for all statistical tests was predetermined at p < 0.05, and significant differences are reported in figures using capital letters to differentiate between groups. Groups with the same letter are not significantly different from one another.

## 3 Results

### 3.1 The rice TLA3 mutant has altered chlorophyll accumulation in leaves

**Figure 1:**
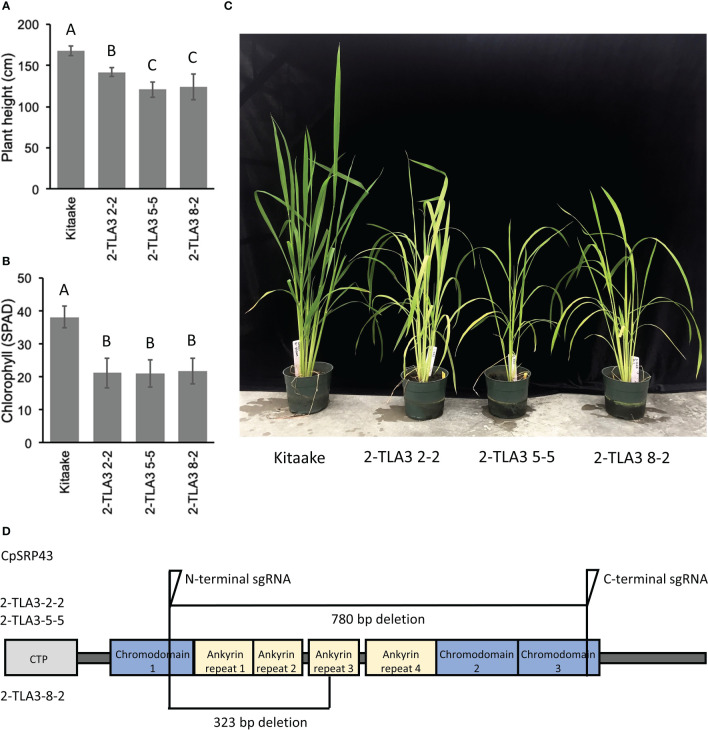
TLA3 mutants have reduced height and chlorophyll content. **(A)** Measurements of plant height for three independent CRISPR/Cas9-generated TLA3 mutant lines as compared to Kitaake wild type (WT). **(B)** Measurements of chlorophyll content for these three mutants, as measured using a SPAD meter. **(C)** Representative images of the homozygous parental line of each of the three mutant lineages characterized in **(A)** and **(B)**, demonstrating reduced stature and a pale green leaf phenotype. **(D)** Protein domain model of validated CRISPR/Cas9-mediated TLA3 mutations generated using two sgRNAs indicating the relative position and size in base pairs of large deletions within CpSRP43. Frameshift mutations occurred in the middle of chromodomain 1 in each line. CTP, Chloroplast targeting peptide.

Rice TLA3 mutations have emerged as the cause of pale-green leaf phenotypes in multiple rice ethyl methane sulfonate (EMS) screens (Lv et al., 2015; Wang et al., 2016; Ye et al., 2018). Likewise, previous EMS and T-DNA screens have also identified putative TLA4 mutations in rice (Zhang et al., 2013; Liu et al., 2016). However, direct comparisons between TLA mutants in rice have been difficult, as these studies were performed in different cultivars, and as EMS-generated mutants may not be complete loss-of-function mutants or may carry additional genetic mutations that contribute to phenotypic outcomes (Wang et al., 2016). To gain a better understanding of the agronomic potential of these TLA mutants, we utilized CRISPR/Cas9 to generate targeted TLA knock-out mutants in the rice cultivar Kitaake. Rice CpSRP43 is encoded by a single gene copy with a single exon. Using CRISPR/Cas9, we generated near-complete gene knockouts of *CpSRP43* in rice (**Figure 1D**) to create TLA3 mutants. In agreement with previous studies, TLA3 mutants had reduced plant height (**Figure 1A, C**). Likewise, these mutants displayed a pale green leaf phenotype and had reduced chlorophyll content (**Figure 1B, C**). These results demonstrate that CRISPR/Cas9 can be successfully implemented to generate targeted TLA-engineered rice plants with phenotypes consistent with the previous studies described above.

### 3.2 A *CpSRP54* duplication occurred in the Poales lineage

**Figure 2:**
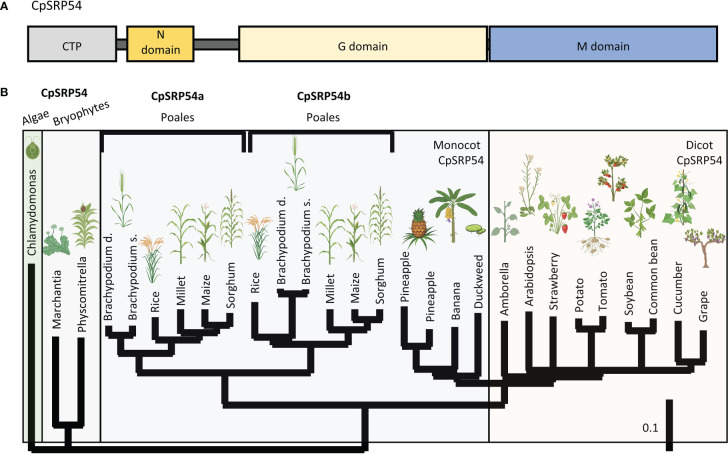
A duplication of CpSRP54 occurred in the Poales lineage. **(A)** Protein domain model of CpSRP54 with CTP (chloroplast targeting peptide). **(B)** Phylogenetic tree of CpSRP54 showing a duplication event in the Poales lineage to produce CpSRP54a and CpSRP54b Created with BioRender.com.

Two previous EMS and T-DNA screens have identified the putative *CpSRP54* gene in rice encoded by LOC Os11g05552 (Zhang et al., 2013; Liu et al., 2016), here called *CpSRP54a*. The canonical CpSRP54a protein structure consists of conserved N, G, and M domains (**Figure 2A**). While CpSRP54 is predicted to be encoded by a single gene copy in algae, mosses, and dicots, a tandem duplication of *CpSRP54a* occurred in an ancestor of the Poales lineage of monocots (here referred to as *CpSRP54b*). This duplication is absent from non-Poales monocot species, such as duckweed and banana, and is present in all sequenced members of the Poales family (**Figure 2B**). To predict whether both genes are involved in the CpSRP pathway, we performed a computational functional gene network prediction using *CpSRP43*, *CpSRP54a*, and *CpSRP54b* as inputs using RiceNet v2 (Lee et al., 2015), which utilizes a large repository of published expression data for rice for its calculations. Network predictions demonstrated that the predicted rice *CpFTSY* was a node shared by all three genes, suggesting that they are all involved in the anticipated CpSRP pathway (**Supplemental Figure 1**). Likewise, CpSRP43 is predicted to functionally interact with the insertase ALB3 (**Supplemental Figure 1**), which has been previously characterized in Arabidopsis as part of the CpSRP pathway (Tzvetkova-Chevolleau et al., 2007). The most direct connections between inputs were shared between CpSRP54a and CpSRP54b, suggesting a strong level of functional overlap. Notably, CpSRP54b has more predicted connections with CpSRP43 than does CpSRP54a (**Supplemental Figure 1**), suggesting that it may play a more important role in CpSRP pathway function than its paralog. Collectively these data suggest that both putative CpSRP54 paralogs are expressed and are predicted to interact with other CpSRP pathway components.

### 3.3 Rice TLA4 and TLA4L mutants can be distinguished by plant height and phenotype

**Figure 3:**
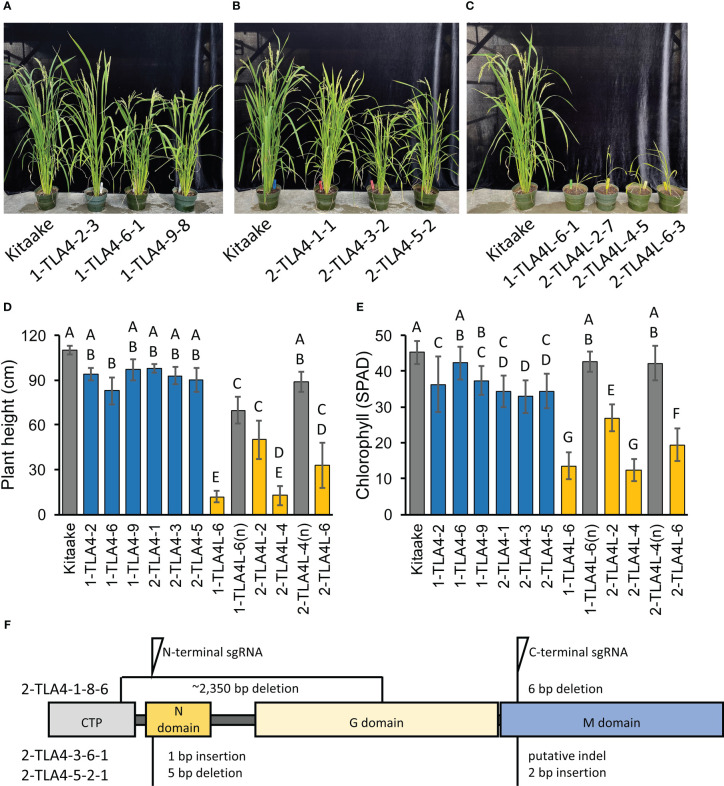
CpSRP54a and CpSRP54b both contribute to leaf chlorophyll accumulation. **(A, B, C)** Representative images of several mutant TLA4 **(A, B)** and TLA4L **(C)** lineages demonstrate reduced plant height and a pale green leaf phenotype as compared to WT. TLA4L mutants display a particularly severe phenotype. **(D)** Plant height of representative TLA4 and TLA4L mutant individuals from independently CRISPR/Cas9-generated lineages. Blue bars represent TLA4 mutants, yellow bars represent TLA4L mutants, and gray bars represent null-segregants (n). Null-segregants show restored WT phenotypes. **(E)** Measurements of chlorophyll content for TLA4, TLA4L, and TLA4L(n) individuals as measured using a SPAD meter. Blue bars represent TLA4 mutants, yellow bars represent TLA4L mutants, and gray bars represent null-segregants (n). **(F)** Protein domain model with CTP (Chloroplast targeting peptide) of validated CRISPR/Cas9-mediated TLA4 mutations generated using two sgRNAs indicating the relative position and size in base pairs of indels within CpSRP54a. All three mutant lines have multiple indels.

To allow for direct comparisons between mutations in CpSRP54a, CpSRP54b, and CpSRP43 in the context of photosynthetic and agronomic traits within the same genetic background, we used CRISPR/Cas9 to generate TLA4 and TLA4L single null mutants and TLA4/TLA4L double null mutants in the Kitaake cultivar (**Figure 3F**). In agreement with previous findings (Shi et al., 2020), we confirmed that TLA4 mutants had reduced plant height, and a demonstrable decrease in chlorophyll content (**Figure 3A-E**). Notably, unlike the findings of Shi et al. (2020), TLA4L rice plants were able to set seed and be propagated (**Figure 3C**). However, compared with the TLA4 mutants, the TLA4L mutants had a greater decrease in both plant height (**Figure 3D**) and chlorophyll content (**Figure 3E**), further suggesting that TLA4L and TLA4 do not perform identical roles within the CpSRP pathway. The inclusion of TLA4L null segregants with restored plant height and chlorophyll content demonstrate that observed phenotypes are due to specific mutations in the target gene and not the result of off-target cutting by CRISPR/Cas9.

Due to the close proximity of *CpSRP54a* and *CpSRP54b* in the rice genome, and the fact that TLA4 mutations reside upstream of the *CpSRP54b* promoter, we hypothesized that the loss of function mutation in TLA4 may have led to altered expression of CpSRP54b, making the dissection of CpSRP54a and CpSRP54b contributions to phenotype difficult in TLA4 mutant lines. To test this, we used quantitative RT-PCR to first confirm the reduced expression of *CpSRP54a* in three independent TLA4 lines (2-TLA4-1-8-6, 2-TLA4-3-6, and 2-TLA4-5-2) compared with WT Kitaake (**Supplemental Figure 2A**). We observed significantly reduced *CpSRP54a* expression in TLA4 mutant lines 2-TLA4-3-6 and 2-TLA4-5-2, and a complete loss of *CpSRP54a* expression in 2-TLA4-1-8-6 (**Supplemental Figure 2A**). Contrary to our hypothesis, no significant reduction in *CpSRP54b* expression was observed in any of the three independent TLA4 mutant lines as compared with Kitaake (**Supplemental Figure 2B**). These results indicate that the phenotypes observed in TLA4 mutants are not due to reduced expression of *CpSRP54b*, but can be attributed to the knockout of *CpSRP54a* alone.

To evaluate the degree to which CpSRP54a and CpSRP54b perform similar functions in the CpSRP pathway, 30 rice plantlets were recovered from tissue culture containing the TLA4/TLA4L sgRNA designed to generate double knockout mutants. However, unlike either single mutant, all putative TLA4/TLA4L double mutant plantlets died shortly after transplanting to the greenhouse. While not definitive, this observation suggests that CpSRP54a and CpSRP54b may contribute additively to chlorophyll accumulation in rice. Combined with the observation that TLA4 mutants do not have reduced expression of *CpSRP54b* yet display significant phenotypes, these results suggest that CpSRP54a and CpSRP54b are both functional proteins with overlapping but not identical roles in rice.

### 3.4 Photosynthetic rate per photon absorbed is increased in TLA mutants

To more precisely determine the relative contributions of CpSRP43 and CpSRP54a to photosynthesis and fitness, a series of experiments were performed to evaluate chlorophyll and carotenoid content, plant height, and photosynthetic rate across physiological development and a range of lighting conditions. TLA4L mutants were not included in this study due to the extremely severe phenotypes associated with the mutations, including dramatically smaller plant size.

First, the pigment content and plant height of three independent lines of TLA3 (2-TLA3-2-5, 2-TLA3-5-5-1-5, 2-TLA3-8-2-3) and TLA4 (2-TLA4-1-8-6, 2-TLA4-3-6, 2-TLA4-5-2) mutants were compared. At five weeks post germination, both the chlorophyll and carotenoid content were significantly reduced in TLA mutants compared to wild-type plants, with TLA3 having lower levels of each pigment than TLA4 (**Supplemental Figure 3**). Similarly, the plant heights of both TLA3 and TLA4 mutants were reduced two weeks after panicle emergence (**Supplemental Figure 3A**). In agreement with previous reports (Lv et al., 2015; Liu et al., 2016; Wang et al., 2016; Shi et al., 2020), chlorophyll a/b ratio was elevated and the ratio of chlorophyll to carotenoids was reduced in TLA mutants (**Supplemental Figure 3F**).

**Figure 4:**
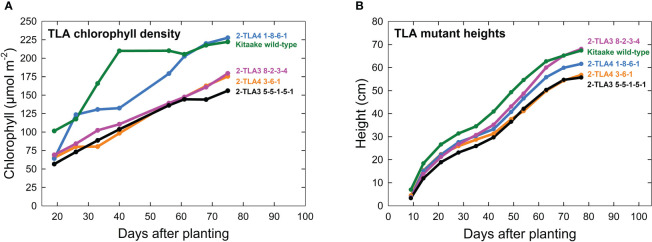
TLA mutants have reduced chlorophyll content and height. **(A)** Plots of chlorophyll content, (y-axis) across time (x-axis) for WT and all mutant lines demonstrate that chlorophyll content increases over time for all lines. **(B)** Most mutants are reduced in height compared to the wild-type.

**Figure 5:**
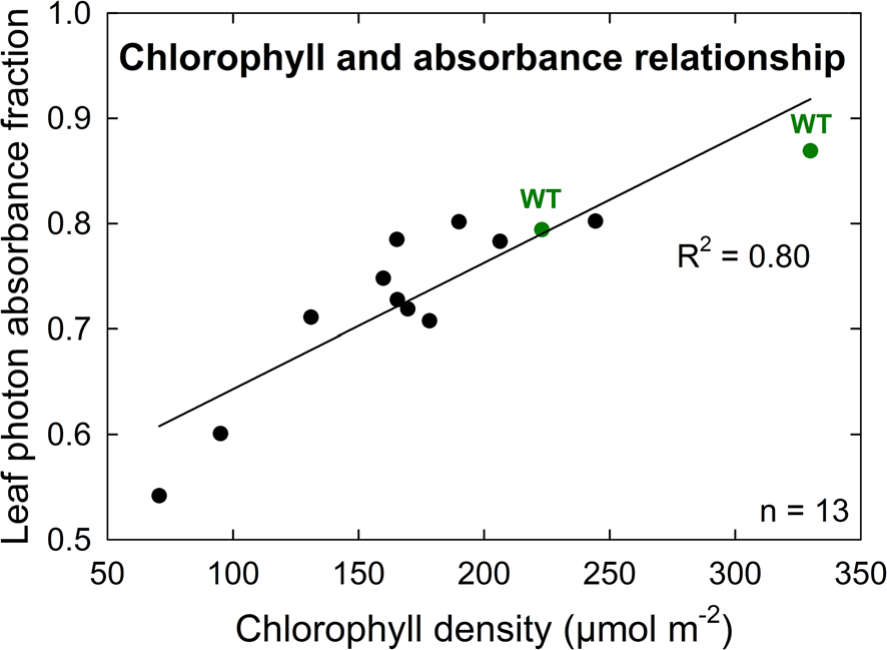
Chlorophyll density is positively correlated with leaf photon absorbance fraction among both wild-type (WT, green) and TLA mutant (black) lines. The high correlation (R^2^ = 0.80) of this relationship implies similar chlorophyll structures and orientations between TLA mutant and WT lines.

A further characterization of TLA phenotypes was performed using two independent mutant lines for both TLA3 (2-TLA3-5-5-1-5-1, 2-TLA3-8-2-3-4) and TLA4 (2-TLA4-1-8-6-1, 2-TLA4-3-6-1).We observed that while chlorophyll concentration increased over time for all lines, rates of accumulation differed between WT and TLA mutant lines (**Figure 4A**). Specifically, while the WT reached a steady chlorophyll concentration after 40 days, all the genetically altered lines continued to increase until heading at 78 days. As expected, plant height steadily increased over time for all lines, but the height of the mutant lines was less than the WT until 60 days after planting (**Figure 4B**). Additionally, we saw that optically measured chlorophyll content was positively correlated with leaf photon absorbance fraction across all lines (**Figure 5**). Collectively, these results confirm earlier observations of decreased height and chlorophyll content during early and mid-development.

**Figure 6:**
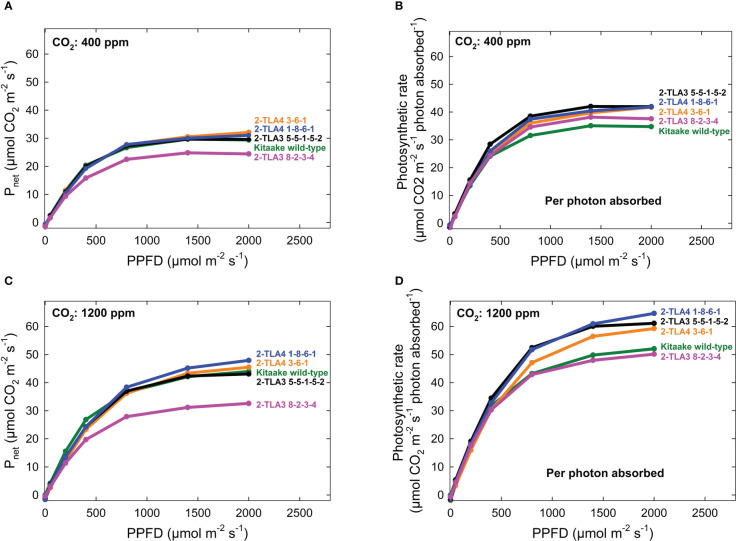
Photosynthetic rates in TLA mutants. The photosynthetic rates for mutant lines are increased compared to the Kitaake wild-type (WT) when accounting for the photosynthetic rate per photon absorbed at 400 ppm CO_2_ **(A, B)**. The relationship is the same at 1200 ppm CO_2_ **(C, D)**. The photosynthetic rate of 2-TLA3 8-2-3-4 (pink) is often less than the photosynthetic rate Kitaake WT (green).

As hypothesized, we also observed that mutant lines exhibited only minimally reduced photosynthetic rate compared to the wild type under standard conditions (**Figure 6A**). When considering the reduced chlorophyll content of all mutant lines, this formally demonstrates that photosynthetic rates per photons absorbed increased in most TLA mutants compared to WT (**Figure 6B**). Furthermore, we demonstrated that the photosynthetic rate across all lines increased at elevated CO_2_, and there was no interaction between increased CO_2_ and mutant lines (**Figure 6C, D**). Significant increases in photosynthesis per photon absorbed in TLA mutants are consistent with the theory that plants overproduce chlorophyll.

## 4 Discussion

Organisms need to safeguard the ability to capture light, which varies diurnally, changes with weather conditions, and can be blocked by shade produced by other plants (Kirst and Melis, 2018). As a genetically encoded strategy to ensure access to light even under suboptimal conditions, photosynthetic organisms often overproduce light-harvesting machinery. The net result is an overproduction of chlorophyll in leaves (Zhao et al., 2020), and at high light intensities there is wasteful absorption of photons by upper leaves, as they far exceed the capacity of the thylakoid membrane for electron transport and the biochemical rate of the carbon reactions of photosynthesis (Kirst et al., 2017; Kirst and Melis, 2018). The excess photons absorbed by upper leaves are used inefficiently for photosynthesis, with an increased fraction of the absorbed light being dissipated as heat (Demmig-Adams et al., 1996; Baker, 2008). By engineering TLA3 and TLA4 mutants with reduced light-harvesting antenna size and chlorophyll content (resulting in more uniform light distribution within the plant canopy) and higher quantum yield compared to WT lines, we show that reduced chlorophyll content is a valid method to improve photosynthetic solar energy conversion efficiency on a whole-plant level. This reduction in chlorophyll content helps to reduce the excess absorption of light by the upper canopy, which increases light transmission to lower leaves, improves photochemical efficiency, and reduces the ensuing wasteful non-photochemical dissipation of excitation energy (Kirst et al., 2018).

Reduced chlorophyll content in leaves can lead to an overall reduction in heat dissipation processes, such as nonphotochemical quenching (NPQ), by limiting the amount of excess energy that is received by the photosynthetic reaction center. A failure in these photoprotective processes can lead to overaccumulation of energy, generation of reactive oxygen species (ROS), and damage to the photosynthetic machinery. However, plants that are too deficient in chlorophyll will capture suboptimal amounts of light energy and suffer a growth penalty as a result. In the case of TLA3 and TLA4 mutants, we observed no detrimental effects in plant growth or appearance throughout the lifecycle of the rice, indicating that this potential reduction had a minimal impact on plant health. We also observed no signs of photoinhibition among the lines and therefore did not measure ROS. However, it is possible that the initial decrease in height observed in TLA3 and TLA4 mutants may be due in part to a decrease in NPQ and associated ROS, which has been shown to decrease leaf elongation in maize (Rodríguez et al., 2002).

Previous research indicates that the CpSRP pathway may behave differently among plant species. In Arabidopsis, single mutants in either *CpSRP43* (*chaos*) or *CpSRP54* (*ffc*) do not drastically reduce chlorophyll accumulation. The CpSRP54 mutant *ffc* first produces yellow leaves, but these eventually recover and produce green leaves by maturity (Amin et al., 1999). A double mutant *chaos/ffc* was required to see a significant and lasting reduction of LCHPs (Hutin et al., 2002). In rice, we observed that TLA4 mutants follow this trend, but TLA4L mutants do not. We also observed that although the engineered TLA3 and TLA4 plants increased in chlorophyll content over time, levels did remain lower than WT until plants were 60 days old.

The potential redundancy between components of this pathway may allow for plants to compensate for the loss of individual components, and is alluded to by the apparent increase in *CpSRP54b* expression in plants with mutated *CpSRP54a* (Shi et al., 2020). This putatively-observed compensation may allow plants with reduced chlorophyll synthesis to reach WT levels given sufficient time, and may indicate that the greatest advantages of these mutants in terms of photosynthetic efficiency and light penetration would occur during early growth, when the most significant reductions in chlorophyll content are observed. Future work is needed to further characterize the change in CpSRP pathway function across developmental stages, which remains largely unexplained.

This study, and other recently published work, also suggests that a Poales-specific duplication of *CpSRP54* has allowed for further evolution of this ancient and essential pathway. In bacteria, the SRP pathway requires the conserved SRP54 protein and an SRP RNA component (Ziehe et al., 2017). In higher plants, the CpSRP pathway maintains the CpSRP54 protein, lacks bacterially-required RNA, and contains a novel, chloroplast-specific CpSRP43 (Ziehe et al., 2017). A recent study in rice found that CpSRP54a and CpSRP54b both independently interact with CpSRP43, though their specific functions in this regard are not yet known (Shi et al., 2020). Specifically, this research has shown that while both CpSRP54a and CpSRP54b appear to colocalize with CpSRP43 in the chloroplast, the phenotypes of the two mutants TLA4 and TLA4L and their impact on gene expression of the remaining members of the pathway are distinct. These observed differences in phenotype for TLA4 and TLA4L mutants suggest that the functions of the two proteins do not strictly overlap. Indeed, it has recently been proposed that these two closely related paralogs may be responsible for transporting different chloroplast proteins into the thylakoid (Shi et al., 2020). Our study confirms that mutations in these two proteins have distinct phenotypes when compared within a common genetic background. While initial heights of TLA4 mutants were reduced compared to WT, they approached normal heights during the grain-fill stage, which suggests that impact on grain yield should be minimal. However, TLA4L mutants showed a more drastic reduction in plant height and chlorophyll content than did TLA4 mutants and never recovered to the stature observed for WT individuals. The difference in severity of observed phenotypes is also consistent with the findings of our CpSRP network analysis, which shows a greater number of predicted connections between CpSRP54b and CpSRP43 than between CpSRP54a and CpSRP43, indicating a potentially more integral role for the former. Previous work also corroborates these findings to suggest that CpSRP54b might be more essential for chloroplast development, as single knockout mutants are significantly smaller and were previously thought to be seedling lethal (Shi et al., 2020). The TLA4L plants generated in this study, however, survived beyond the seedling stage, although they remained severely stunted, developmentally delayed, and had poor seed set.

The prior study conducted by Shi et al. (2020) compared an EMS mutation of TLA4 in the cultivar IR64 and a CRISPR mutation in TLA4L in the cultivar Kitaake, preventing a comparison of TLA4 and TLA4L function in an isogenic background, and making the development and analysis of a double mutant challenging. Finally, it is worth noting that our study utilizing CRISPR/Cas9 enabled a direct test of the viability of a TLA4/TLA4L double mutant, which is not feasible by genetic crossing, as the two genes are located at the same genetic locus. In our study, we failed to produce any double TLA4/TLA4L mutants, suggesting that CpSRP54a and CpSRP54b collectively contribute to an indispensable part of plant development. The large number of overlapping functions between these proteins as shown in our CpSRP network analysis substantiates that our lack of double mutants was likely not due to CRISPR/Cas9 methodological challenges but is instead indicative of plant lethality in double mutants. Collectively, the phenotypic differences among TLA mutants in both the present and prior studies suggest that CpSRP genes likely vary in suitability as targets for plant engineering, and that care must be taken in choosing the target to avoid detrimental phenotypes.

In the wild, plants maximize light absorption through synthesis of large light-harvesting antenna, which makes individual plants more competitive by capturing more photons than neighboring plants (Kirst and Melis, 2018). However, in monoculture agriculture this normally beneficial trait leads to decreased light use efficiency and reduced yield potential because the top leaves absorb about 95% of the photons and the remaining leaves are light-starved. In theory, increased light transmission to lower leaves means more equal light distribution among leaves, especially in plants with a planophile (horizontal) leaf architecture, where excessive light absorption by the top-most leaves results in non-uniform light distribution. More uniform distribution of light among leaves should lead to increased canopy photosynthesis and yield. This study demonstrates the potential for this novel yet counterintuitive approach toward crop improvement *via* the knockout of essential genes and paves the way for work in other crop species. Future investigations involve expanding this work to larger scale field studies under multiple planting densities to show community-level improvements.

## Data availability statement

The original contributions presented in the study are included in the article/**Supplementary Materials**. Further inquiries can be directed to the corresponding author.

## Author contributions

Conceptualization, DC-D, BB and DC; methodology, DC, NL, SZ, LM; formal analysis, DC, NL; data curation, RK; writing—original draft preparation, DC, DC-D, BB, NL, ME, RK; supervision, DC-D, BB; project administration, DC-D. All authors contributed to the article and approved the submitted version.
