# Supplementary figures and images for: Photosynthesis in rice is increased by CRISPR/Cas9-mediated transformation of two truncated light-harvesting antenna
Source: Front Plant Sci. 2023 Jan 19;14:1050483. doi: 10.3389/fpls.2023.1050483 (PMC9893291; doi:10.3389/fpls.2023.1050483)

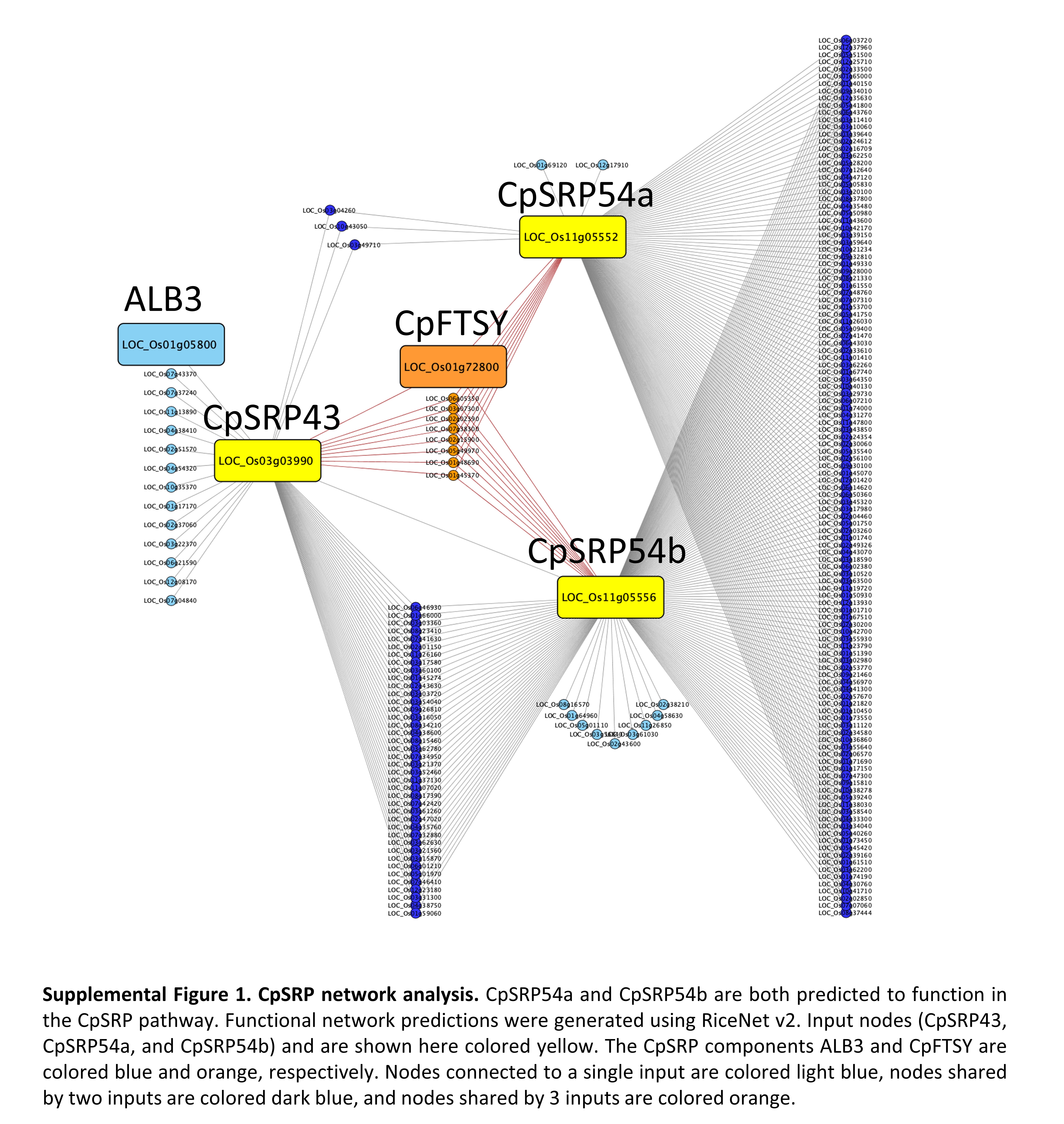

Supplement: Supplementary file 1 [file Image_1.jpeg]

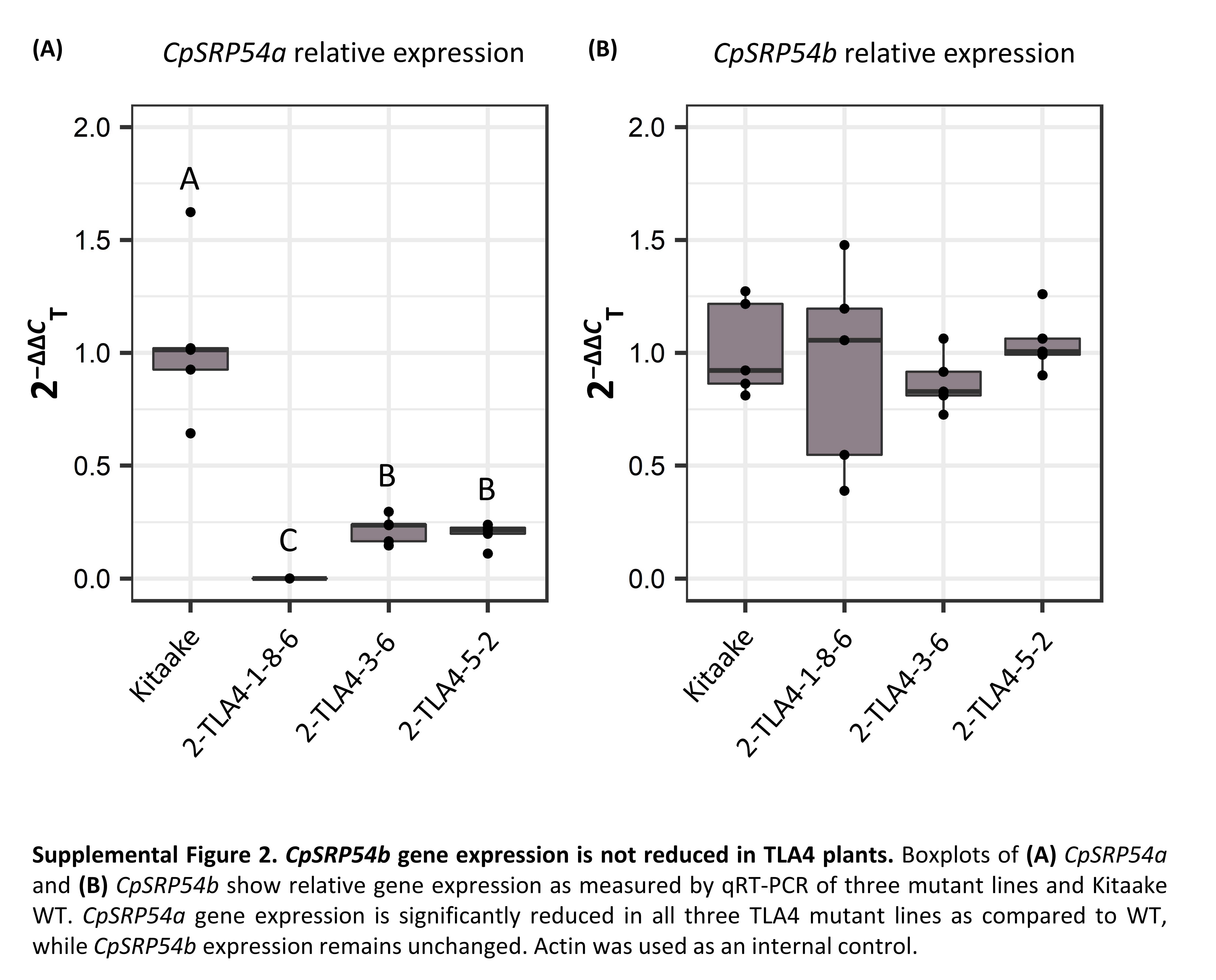

Supplement: Supplementary file 2 [file Image_2.jpeg]

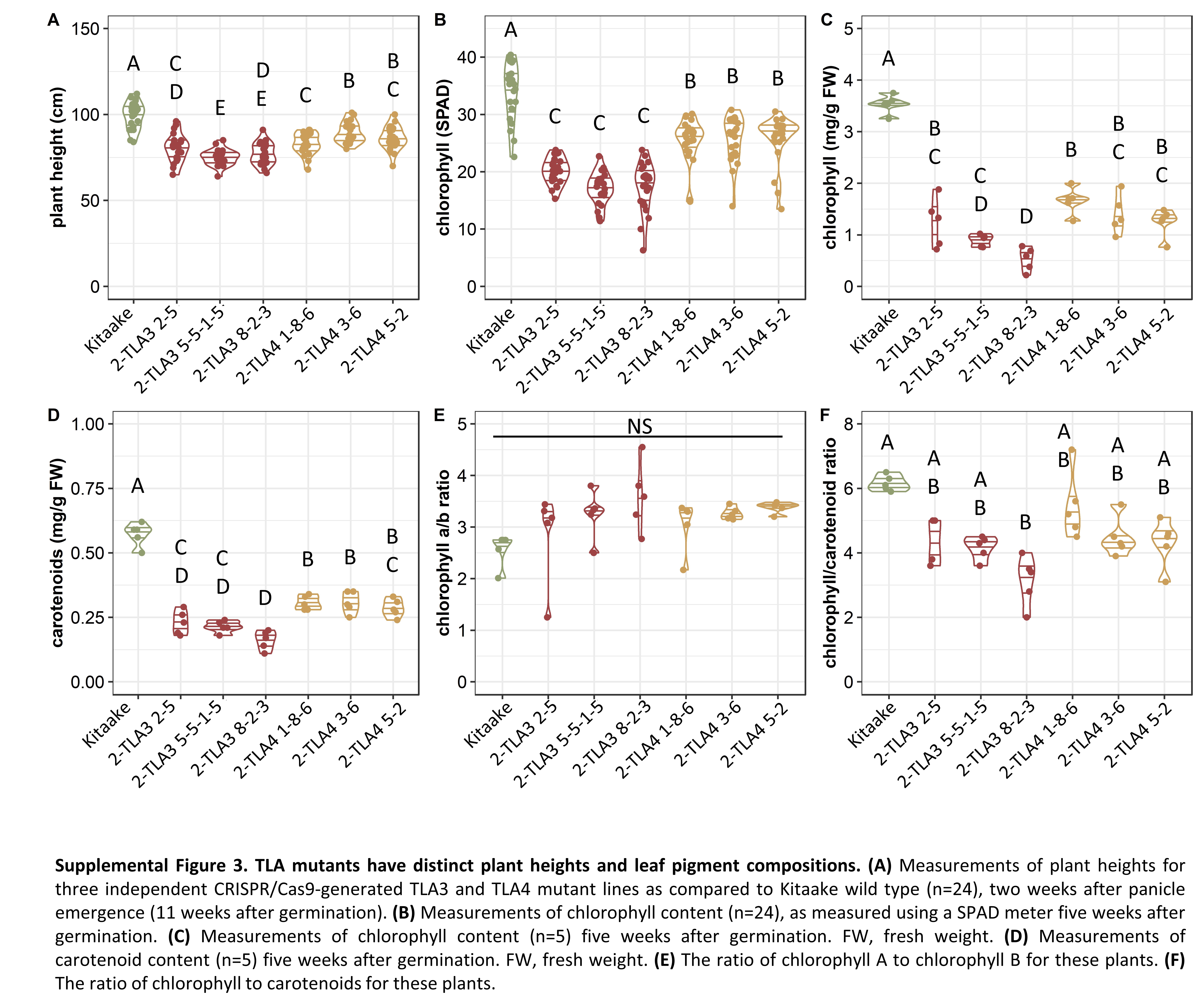

Supplement: Supplementary file 3 [file Image_3.jpeg]
